# Study on flame fractal characteristics of coal dust explosion at the decompression position of Hartmann tube

**DOI:** 10.1038/s41598-024-83040-z

**Published:** 2025-04-08

**Authors:** Zemiao Yang, Ke Gao, Yinhui Wang

**Affiliations:** 1 College of Safety Science and Engineering, Liaoning Technical University, Huludao, 125105 China; 2 Key Laboratory of Mine Thermodynamic Disasters and Control of Ministry of Education, Liaoning Technical University, Huludao, 125105 China; 3 State Key Laboratory of Coal Mine Disaster Prevention and Control, Fushun Liaoning, 113122 China; 4 Ordos Institute of Liaoning Technical University, Ordos, 017004 China

**Keywords:** Coal dust explosion, Schlieren image, Flame dynamics, Fractal dimension, Energy science and technology, Engineering

## Abstract

The explosion of coal dust is powerful and has a wide range of spread, which can easily cause mass death and injury. In this paper, the flame microstructure and propagation characteristics of coal dust explosion are recorded by high-speed photography schlieren technique. The experimental results show that the flame shape of Hartmann outlet is mushroom cloud with obvious fractal characteristics, and the fractal dimension can measure its complexity. When the particle size of coal dust is constant, the fractal dimension increases first and then decreases with the increase of concentration. When the coal dust particle size is less than 53 μm and the initial concentration is 300 g/m^3^, the flame fractal dimension reaches the maximum value of 1.71. The fractal dimension decreases with the increase of coal dust particle size. The coal dust explosion flame has a cellular structure, and its instability is caused by the combined action of hydrodynamic instability and unequal diffusion instability. These instabilities make the flame front structure develop from wrinkles to cell bodies and change continuously. This study can reveal the complexity of coal dust explosion flame propagation at the outlet position, and provide a new perspective for in-depth understanding of coal dust explosion propagation behavior.

## Introduction

With the continuous development of society, coal still occupies a dominant position in China ‘s energy system. In the process of coal mine production, the potential safety hazards of coal dust explosion cannot be ignored^1–3^. The process of coal dust explosion is accompanied by the rapid propagation of flame, the structure of turbulent flame has an important impact on the propagation of coal dust explosion. Therefore, the flame propagation law and flame propagation form are crucial for comprehensively investigating the mechanism of coal dust explosion.

In recent years, both domestic and international scholars have conducted extensive research on the influence of coal dust particle size^4–6^, mass concentration^7–9^, volatile content^10–13^, ignition energy, and other factors^14,15^ on the characteristics of coal dust explosions. Tan et al.^16^ used a 20 L spherical explosion test device to compare and analyze the explosion pressure characteristics of micro-nano pulverized coal particles at five concentrations. Nie et al.^17^ analyzed the research progress of the mechanism of pulverized coal explosion at the macro and micro levels, and proposed a four-stage coal dust explosion mechanism. Zhou et al.^18^ studied the effects of pulverized coal type and methane content on pulverized coal/methane mixed explosion by using a gas-powder two-phase explosion test system. Jia et al.^19^ investigated how particle size and concentration of coal dust affect the explosion characteristics of gas-coal mixtures. The results indicated that under constant mass concentration of coal dust, there was a consistent decrease in explosion pressure as particle size increased. Abbas et al.^20^ introduced an innovative method for experimentally determining both the minimum explosive concentration in flammable dust clouds and the lower limit for mixture explosions. Ban et al.^21^ discovered that as ignition energy increased, the combustion rate of coal dust with varying volatile content also increased, leading to more intense deflagration reactions. Additionally, as the concentration of coal dust increases, the heat transfer efficiency is enhanced, and the flame diffusion speed accelerates.

With the extensive and in-depth study of dust explosion mechanism and disaster evolution process, flame propagation process and flame microstructure have attracted the attention of scholars at home and abroad and a lot of research has been carried out.

Yu et al.^22,23^ found that the addition of H_2_ can improve the brightness of the flame and improve the continuity of the flame front. Pei et al.^24^ compared the explosion characteristics of methane/pulverized coal and methane/graphite powder and found that when the methane concentration increased to 9%, the combustion flame shape of the two mixed systems changed from irregular to spherical. Zheng et al.^25^ experimentally studied the change process of the ignition position dominating the flame structure in the open pipe. Li et al.^26^ studied the effect of hydrogen content on the ignition characteristics of methane-hydrogen fuel by experimental methods. It was found that with the increase of hydrogen content, the diffusion thermal instability and hydrodynamic instability were significantly enhanced, resulting in irregular changes in flame structure. Zhang et al.^27^ detected the morphology of the combustion region and the preheated region and recorded the flame propagation characteristics. The results showed that there was a significant difference in the morphology of the combustion region and the preheating region. Roy and Sujith^28^ explain the change of internal cut-off frequency caused by intermittent dissipation through the coarse-grained approach based on moments of the dissipation and the fine-scale analysis method by adopting the multifractal formalism. The upper limit of fractal dimension is corrected, and the influence of intermittent dissipation on the fractal dimension of low turbulence premixed flame is clarified. Yi et al.^29^ used high-speed schlieren photography to study the flame microstructure and propagation characteristics of methane explosion, and used fractal dimension to clarify flame instability. With the development of modern computer and image processing technology, a high-speed and non-interference method has been provided for the study of combustion and explosion mechanism^30^. At the same time, digital image processing is used to quantitatively analyze the flame front and internal structure, so as to study the flame propagation mechanism of coal dust explosion.

This study focuses on the key aspect of the location of the flame generated by coal dust explosion at the Hartmann outlet. The flame generated by the coal dust explosion propagates upwards from the closed end of the bottom of the Hartmann tube, the outlet serves as a communication point between the tube and the outside atmosphere. The propagation pattern of the outlet position determines the way and speed of the explosion energy released to the outside, which affects the scope and extent of the explosion damage. Therefore, in this paper, the flame schlieren images of coal dust explosion at Hartmann outlet were recorded by high-speed camera, and the temporal and spatial evolution characteristics of flame under different coal dust concentration and particle size are analyzed by fractal theory. In this way, the propagation law of coal dust explosion flame at the special position of the outlet can be more accurately revealed, and a new perspective can be provided for in-depth understanding of the propagation behavior of coal dust explosion.

## Experiments

### Experimental setup

**Fig. 1:**
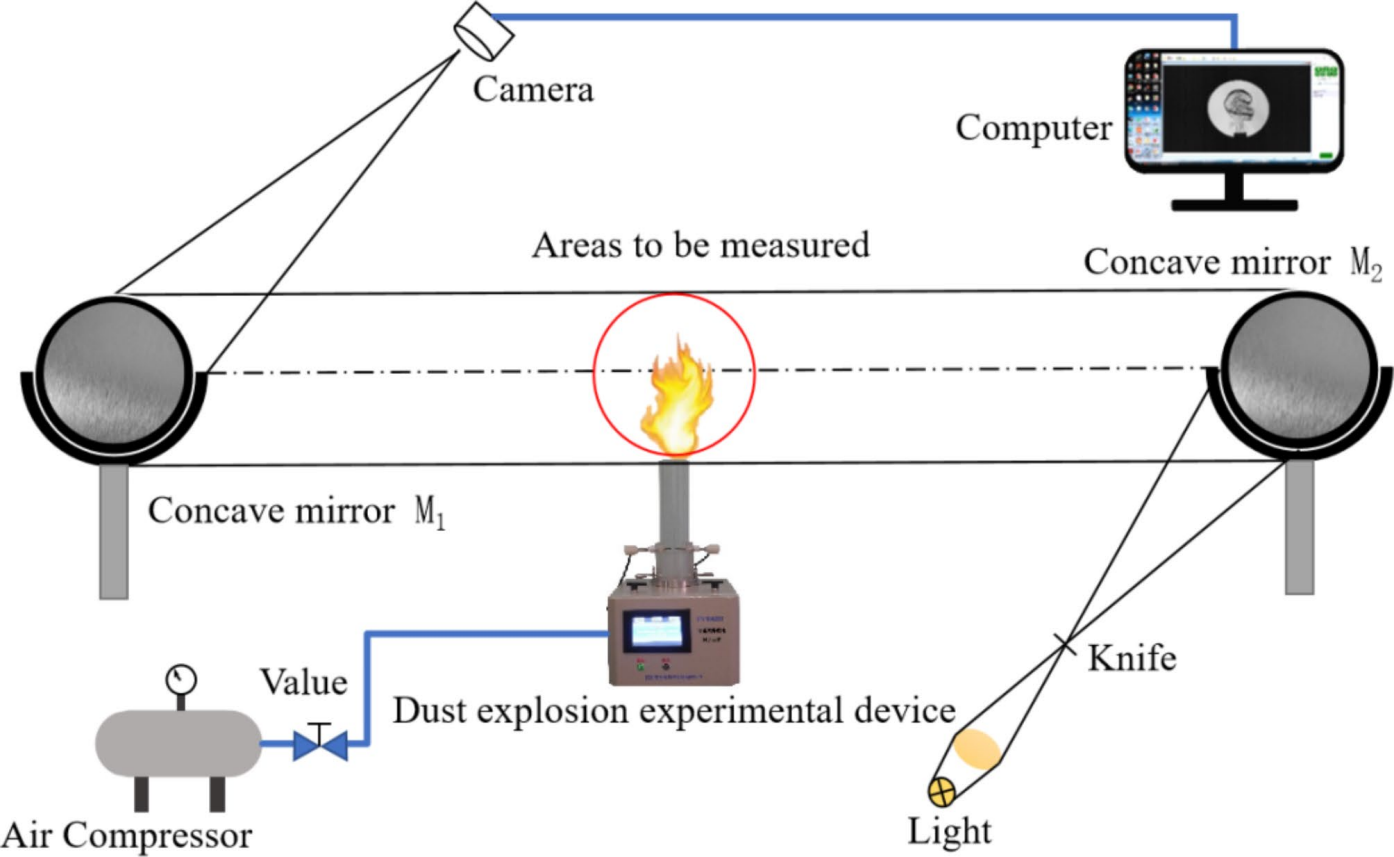
Diagram of experimental device.

The propagation characteristics of coal dust flames were investigated using a schlieren imaging system and a dust explosion characteristic testing device. Figure 1 illustrates the dust explosion experimental setup comprising a vertical combustion glass tube, an ignition system, a high-pressure dust dispersion system, an intake system, a high-speed camera, a synchronous control system, and a data acquisition system. The vertical combustion tube is made of quartz glass with dimensions: diameter of 80 mm, wall thickness of 5 mm, height of 310 mm, volume of 1.2 L, and an open top. The ignition system is positioned 100 mm above the base of the combustion tube, and ignition is achieved through electrode discharge. The total length of the ignition electrode measures approximately 110 mm, with a radius of curvature at the electrode tip ranging from 0.001 to 0.004 mm^− 1^, and a distance of 6 mm between the two electrode tips. The ignition energy is set at 7.5 J. Two types of pulverized coal with varying particle sizes (53 –75 μm, less than 53 μm) were sifted through standard sieves 200 mesh and 300 mesh. The pulverized coal was uniformly distributed at the base of the tube, and high-pressure powder injection with a pressure of 250 kPa was used to disperse the coal dust. This process resulted in the formation of uniform coal dust clouds with initial concentrations of 200 g/m^3^, 250 g/m^3^, 300 g/m^3^, 400 g/m^3^, 450 g/m^3^, 500 g/m^3^, 600 g/m^3^, 700 g/m^3^ in the combustion tube respectively. The concentration is defined as the ratio of mass weight of coal dust introduced in the tube to tube volume being 1.2 L. The intake system primarily comprises an air compressor and gas storage chamber. For the experiment, the VEO 340 S high-speed camera from Phantom Company was utilized. The synchronous control system is linked to the data acquisition system, overseeing the test process through both the intake and ignition systems. The high-speed camera is synchronized with the data acquisition system. Upon successful data acquisition, the information is then transferred to the computer for storage. Schlieren imaging was utilized to observe the temporal and spatial evolution characteristics of the flame at the outlet position.

### Experimental materials

**Fig. 2:**
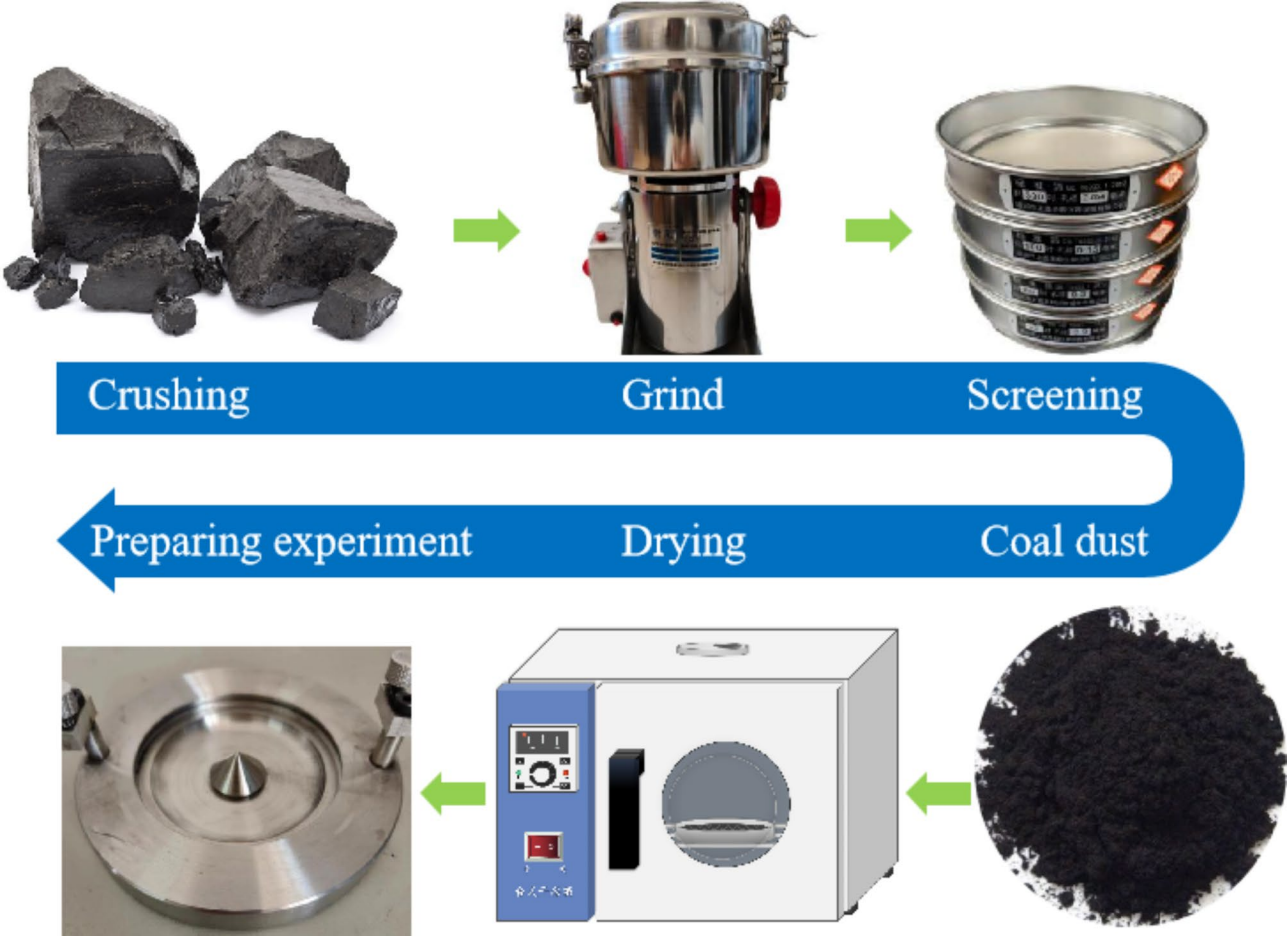
Preparation of experimental materials.

To investigate the flame propagation traits of coal dust explosions, coal samples underwent crushing and screening. Standard sieves of 200 mesh and 300 mesh nominal diameters were utilized for screening the coal dust. Following screening, two types of pulverized coal particle samples with varying sizes were acquired. The particle size distributions 53–75 μm (200 mesh) and less than 53 μm (300 mesh). The preparation process of the experimental material is depicted in Fig. 2. Once the coal dust is dried and cooled to room temperature, it is enclosed in a labeled sealed bag containing information on name and particle size, then stored in a drying dish.

**Fig. 3:**
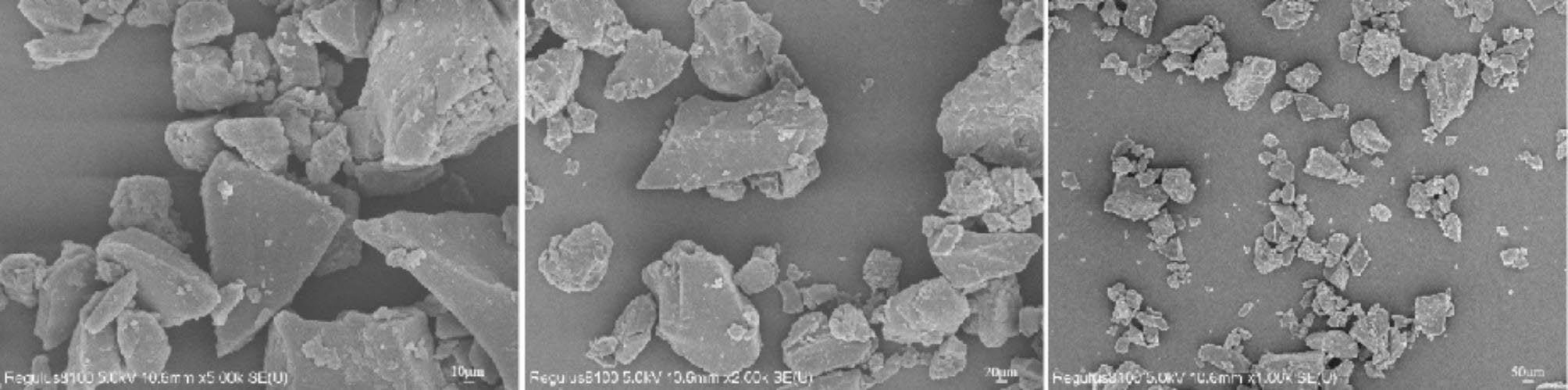
Scanning electron microscopy of the coal particle.

To analyze the surface morphology of coal dust in more detail, a Hitachi Regulus 8100 scanning electron microscope SEM was employed to examine coal dust particles sized at 300 mesh. Figure 3 shows that coal dust exhibits irregular aggregation, suggesting inherent specific aggregation properties. Coal dust particles have irregular shapes with distinct edges and corners, intricate surface structures, and numerous micropores and fissures dispersed throughout. The particle size distribution of coal dust particles observed through scanning electron microscopy primarily falls below 53 μm, consistent with the particle size determined through standard sieve screening.

The explosion of coal dust cloud is essentially the explosion of combustible gas emitted by the thermal release of coal dust and the combustion process of fixed carbon. In order to study the influencing factors of coal dust explosion intensity, the moisture, ash and volatile content in coal samples were measured and calculated, and the industrial analysis of coal samples was obtained as shown in Table 1.

**Table 1:** Industrial analysis of coal samples.

Sample	Proximate analysis W_ad_ /%			
	M_ad_	A_ad_	V_daf_	FC_ad_
Coal samples	3.93	3.03	33.29	59.75

M_ad_: moisture content; A_ad_: ash content; V_ad_: volatile content; FC_ad_: fixed carbon content.

## Results and discussions

### Fractal dimension of coal dust explosion flame

#### Flame shape fractal characteristics

**Fig. 4:**
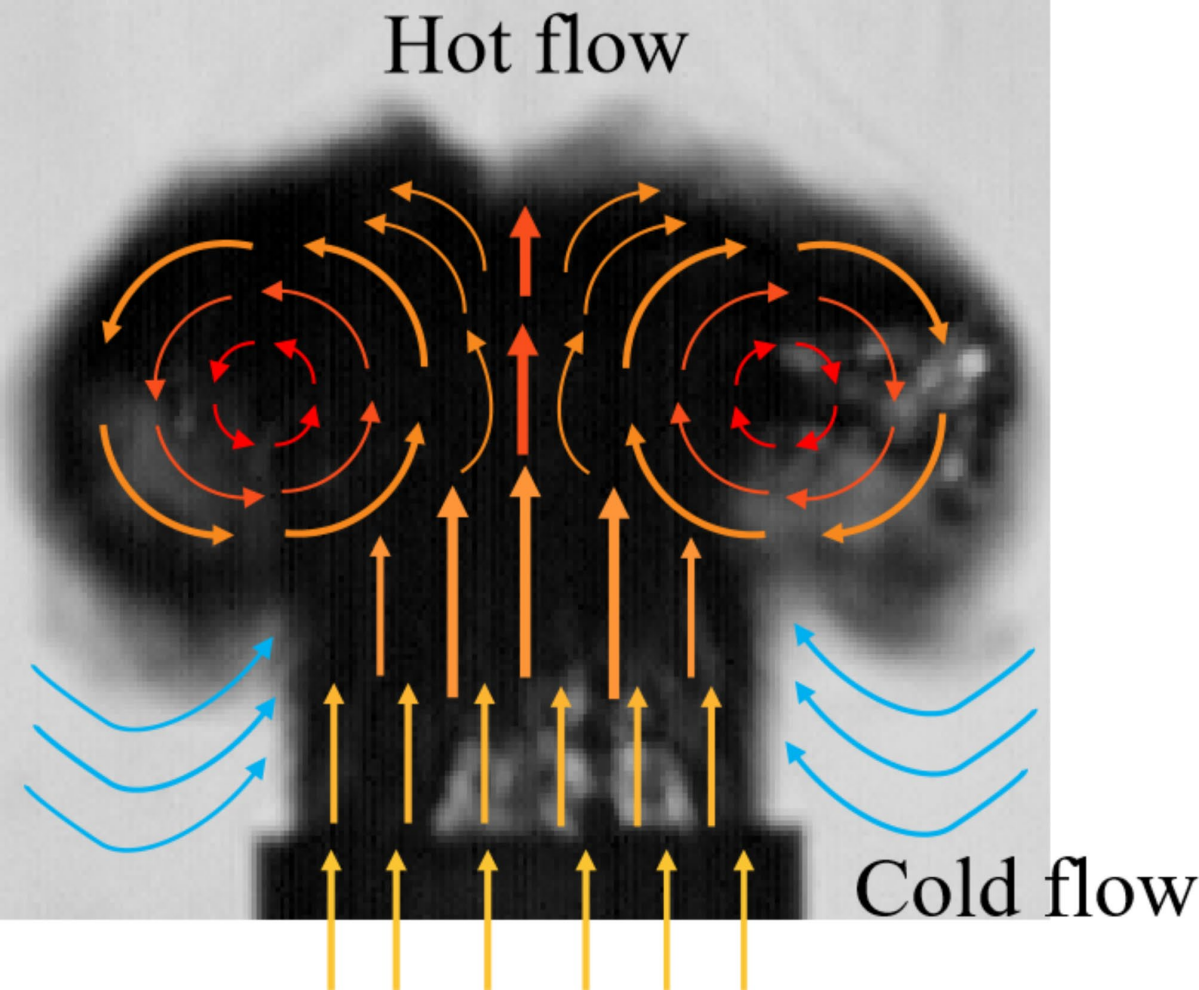
The formation of a mushroom cloud shaped flame.

When the flame reaches the outlet position of the vertical combustion tube, the flame morphology changes significantly, and the flame spreads from the confined tube to the surrounding free space. In this diffusion stage, the hot gas gradually spreads and settles in all directions due to the density difference and buoyancy effect. At the same time, the cold airflow at the bottom rises rapidly under the strong heating and entrainment effect of the hot airflow. This convection makes the flame shape present a typical mushroom cloud shape, as shown in Fig. 4. The central thermal airflow rises, and the peripheral cold airflow surrounds and supplements upward, forming a distinct boundary and dynamic structure.

When studying the flame image in the shape of mushroom cloud produced by the coal dust explosion at the Hartmann outlet position, it was found that the flame has various shapes and its boundaries were complex and changeable. From the perspective of the intersection of mathematics and physics, this complex and changeable boundary feature can be regarded as a fractal structure with statistical significance. As an extremely effective theoretical tool in dealing with complex geometric shapes and self-similar structures, fractal theory can be accurately applied to the study of flame morphological characteristics and explore the effects of different concentrations and particle sizes on coal dust explosion flames. In the whole combustion process of the flame, the flame surface morphology is always in dynamic change, which shows a high degree of complexity and significant fractal characteristics. The continuous evolution of flame morphology makes its structure and morphology show unique self-similarity and scale-free at different scales. This self-similarity means that observing the local structure of the flame at different magnifications will find that it has similar morphological characteristics to the overall structure. The scale-free property indicates that the morphological characteristics of the flame do not depend on a specific scale, and can maintain similar complexity in a wide range of scales. Therefore, the flame morphology at the Hartmann outlet position can be regarded as a fractal structure with statistical significance.

Through the use of image processing technology and mathematical calculation methods, the key parameters such as fractal dimension and fractal characteristics of the flame surface are analyzed in depth, and the morphological characteristics and combustion behavior of the flame can be understood more comprehensively and carefully. As an important index to measure the complexity of flame shape, fractal dimension can reflect the fineness and irregularity of flame structure. The larger the fractal dimension is, the more complex and irregular the flame structure is. Conversely, the smaller the fractal dimension is, the simpler and more regular the flame structure is. Furthermore, the inherent law and mechanism of flame combustion process are revealed.

#### Fractal dimension of flame at outlet position

The fractal dimension of an object depends on its characteristics and the research purpose. When an object exhibits typical fractal features like self-similarity (including statistical self − similarity) and scale-free properties, various methods can be used to quantify it, such as Hausdorff dimension, information dimension, similarity dimension, correlation dimension, capacity dimension, spectrum dimension, and Lyapunov dimension^31^. Among these dimensions, the Hausdorff dimension stands out due to its broad applicability and significance in analyzing the fractal characteristics of objects. It is commonly employed for this purpose.

In Euclidean geometry for regular geometric shapes like triangles, squares, and circles there exists a specific mathematical relationship between perimeter and area as follows:

1 $$P \propto {A^{1/2}}$$

*P* represents the perimeter of the figure, while *A* denotes its area.

The conventional Euclidean geometric measurement approach is inadequate for irregular shapes like natural islands and micro-cracks in materials. Mandelbrot introduced fractal theory for intricate geometric forms, highlighting a correlation between the fractal dimension (*D*_*f*_) of an irregular figure’s boundary and its perimeter and area, expressed by formula 2.

2 $${P^{1/{D_f}}} \propto {A^{1/2}}$$

Here, *D*_*f*_ represents the fractal dimension of the boundary line of an irregular figure, and the logarithm of Eq. 2 is derived.

3 $$\ln P=C+\left( {{{{D_f}} \mathord{\left/ {\vphantom {{{D_f}} 2}} \right. \kern-0pt} 2}} \right)\ln A$$

In Eq. 3, a ln*P*-ln*A* relationship diagram is plotted, where *C* represents a constant. The slope of the straight line in the graph corresponds to the fractal dimension, which is twice the slope value.

**Fig. 5:**
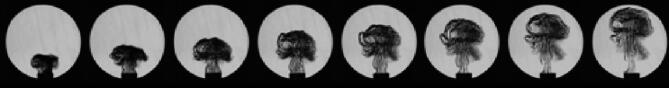
The schlieren diagram of the flame propagation process of coal dust explosion with a concentration of 350 g/m^3^ at the tube outlet, particle size less than 53 μm.

The flame at the outlet position of the Hartmann tube exhibits fractal characteristics of statistical self-similarity. The focus of study is primarily on the mushroom cloud-shaped flame. The “Slit Island Method”^32,33^ can be utilized to depict its fractal features. This method is also known as the area-perimeter method. Initially, calculate the perimeter *P* and area *A* of each island (closed curve) on every isosurface. Subsequently, take logarithms of both perimeter and area; plot ln*P*-ln*A* distribution points on a coordinate system and perform linear fitting on these points. Finally, calculate to determine the fractal dimension. The flame schlieren diagram of coal dust explosion at the tube outlet position at different times is shown in the Fig. 5.

#### Relationship between fractal dimension and coal dust concentration and particle size

**Fig. 6:**
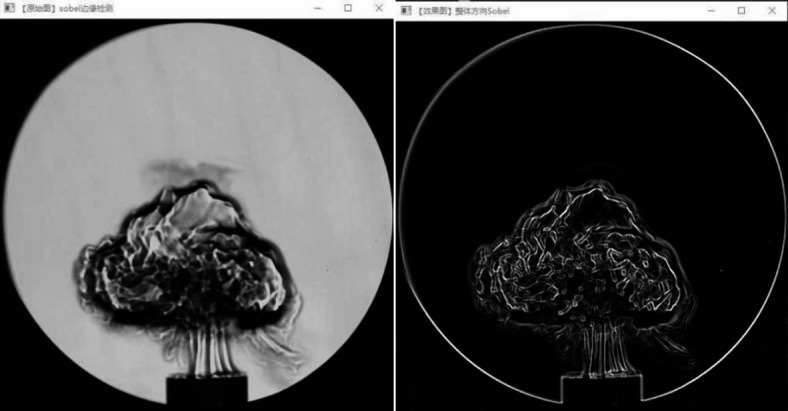
Overall direction Sobel edge detection Schlieren picture.

Based on image recognition method and fractal dimension theory, the flame propagation images captured by high-speed camera are analyzed. Firstly, the image preprocessing technology is carried out to convert the color image into the grayscale image. According to the Eq. 4, the RGB value of each pixel in the image is accurately converted into the grayscale value, so as to effectively reduce the amount of data. The image is further processed in a binary process. By scientifically setting the appropriate threshold, if the gray value of each pixel in the image is higher than the threshold, the pixel is judged to belongs to the flame-related region. Conversely, if the gray value of each pixel is lower than the threshold, it is identified as the background area. It can effectively eliminate the redundant information in the image and highlight the fine structure of the flame. As shown in Fig. 6, the flame edge of coal dust explosion is extracted by Sobel operator, and the contour of flame front is successfully separated from the image. In this way, the flame shape can be separated from the complex background, and finally the flame edge can be effectively extracted.

4 $$Gray=0.299R+0.587G+0.114B$$

**Fig. 7:**
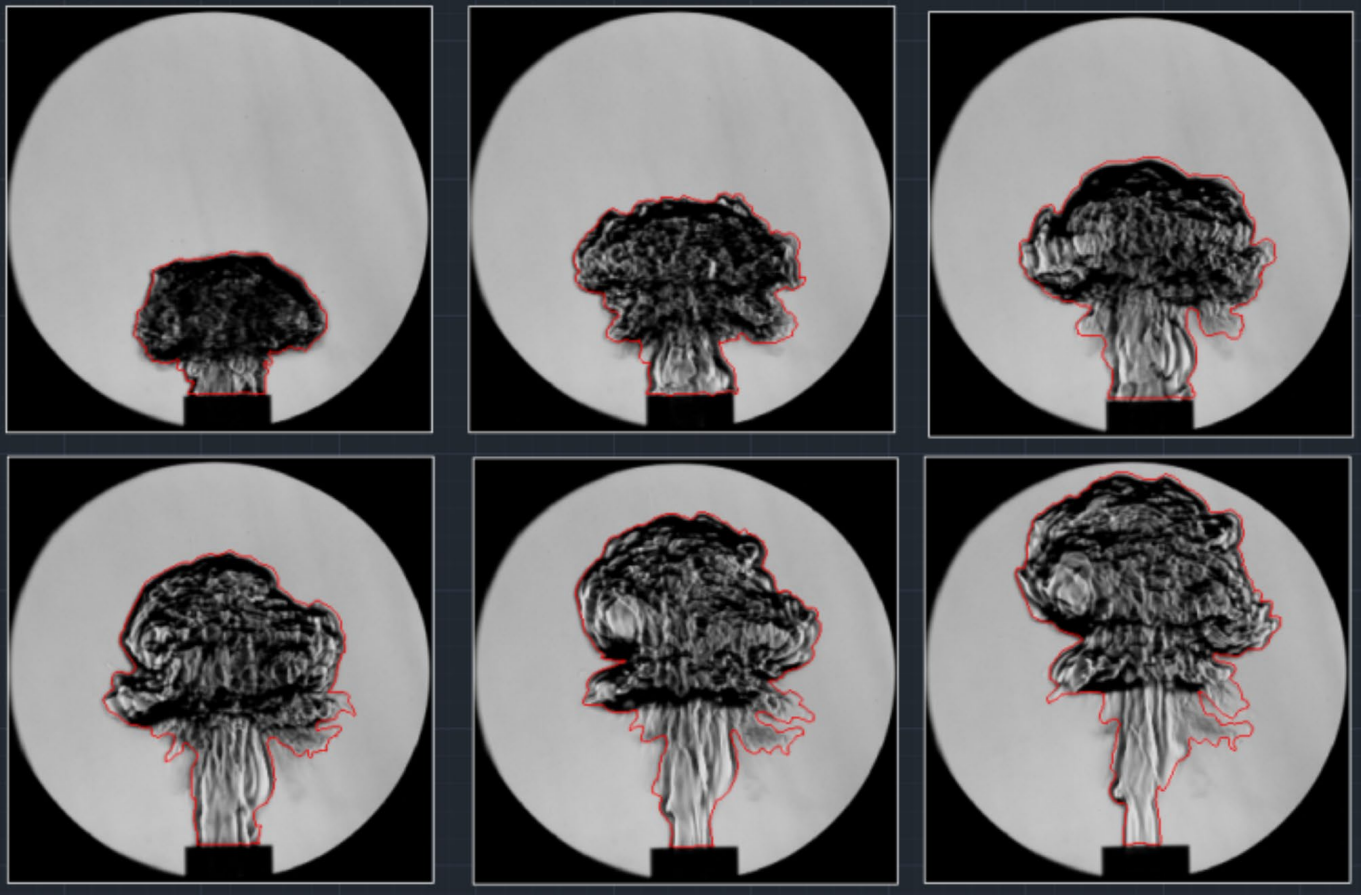
Extraction of flame contour of coal dust explosion.

In the schlieren image, the flame density starkly contrasts with its surroundings, this indicates that the coal dust flame shape possesses an independent, complete, and enclosed boundary at every moment. The perimeter and area formed by these boundaries reflect the fractal characteristics of the flame. Through analyzing a series of coal dust flame images under varying coal dust particle sizes and concentrations. Beginning from when the mushroom cloud’s flame shape is fully developed, depicted in Fig. 7, accurately identifying the flame boundary allows for extraction of data on both its area and perimeter.

**Fig. 8:**
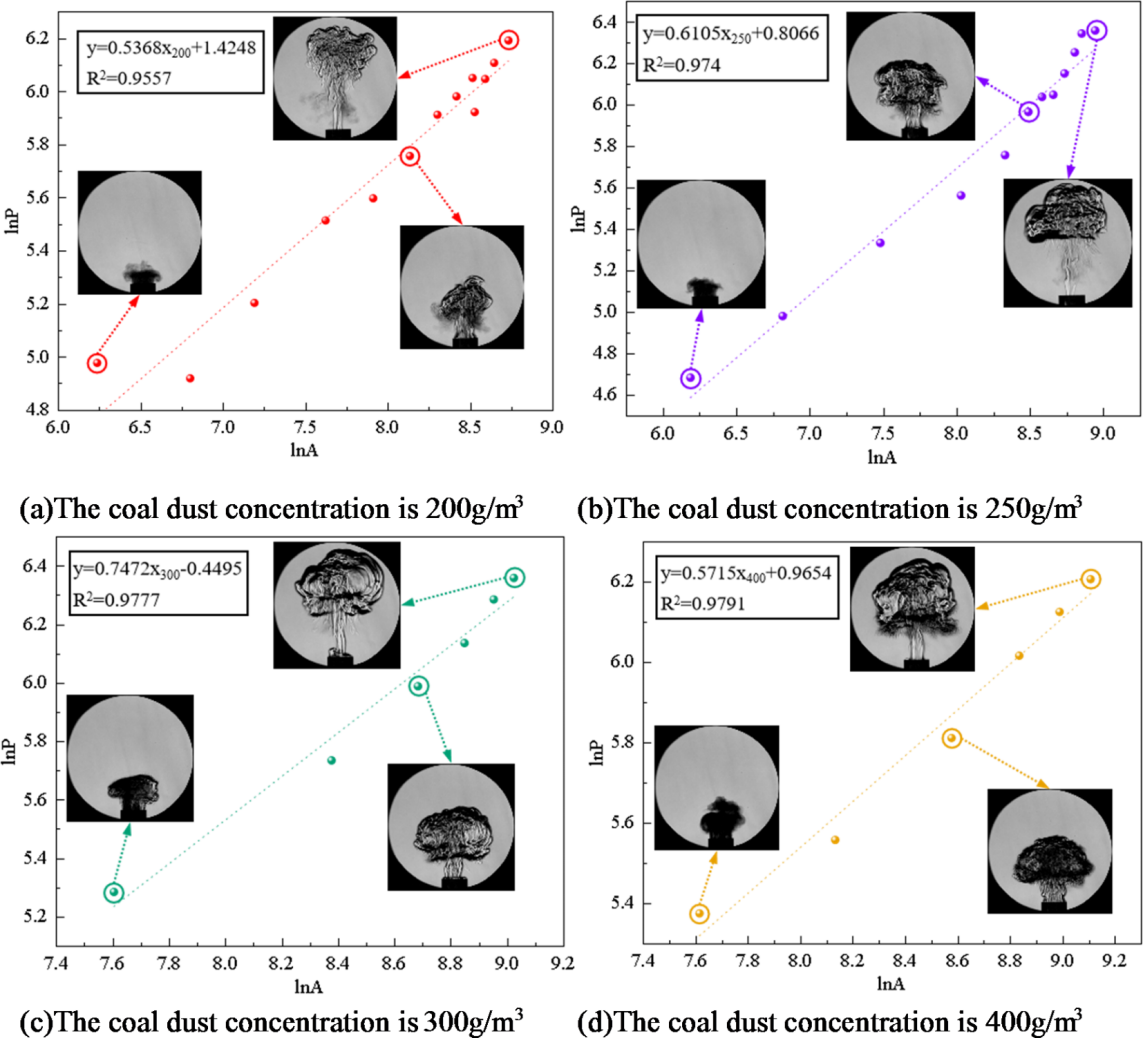

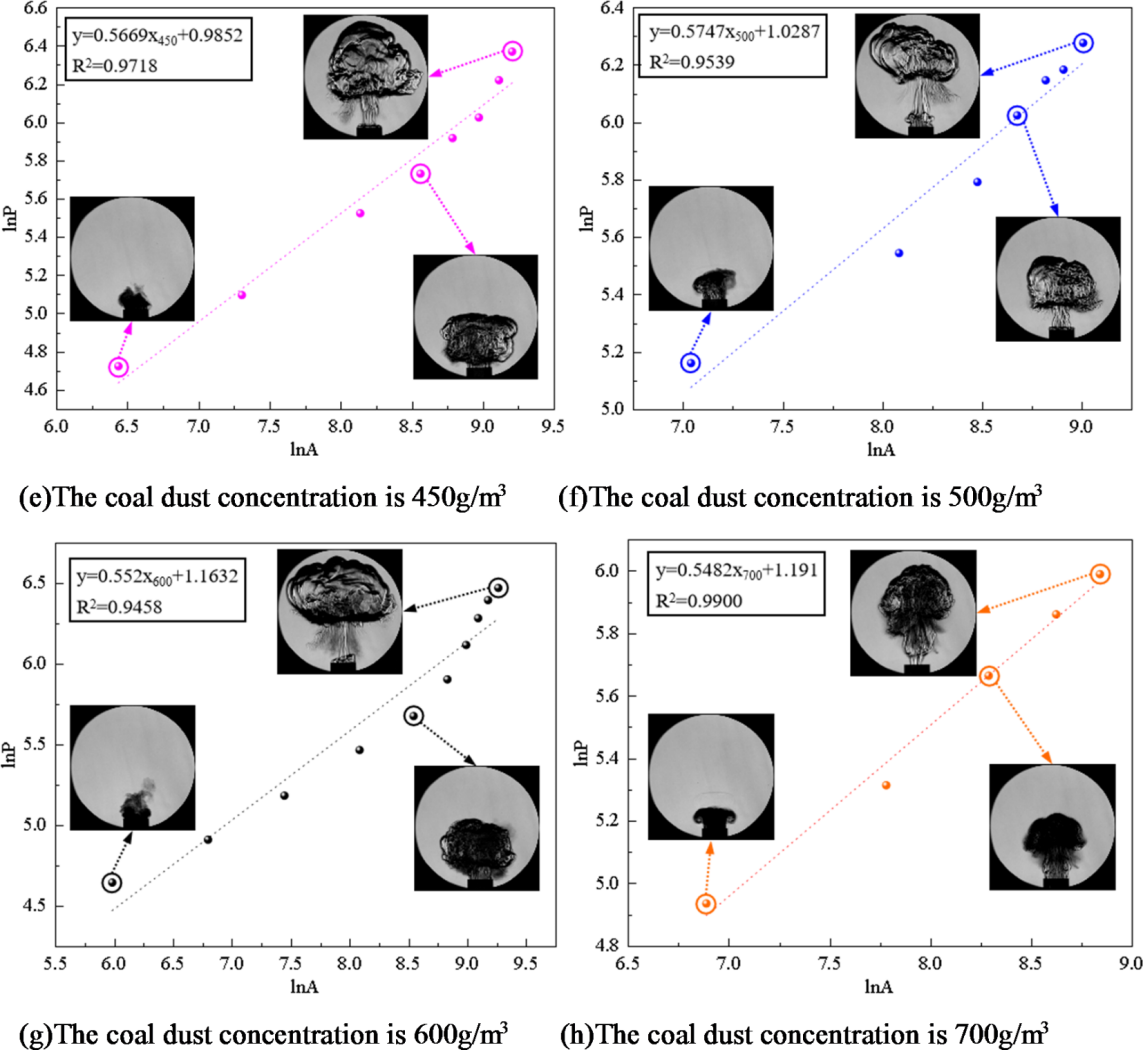
Flame fitting equation of coal dust particle size 53–75 μm at different concentrations.

**Fig. 9:**
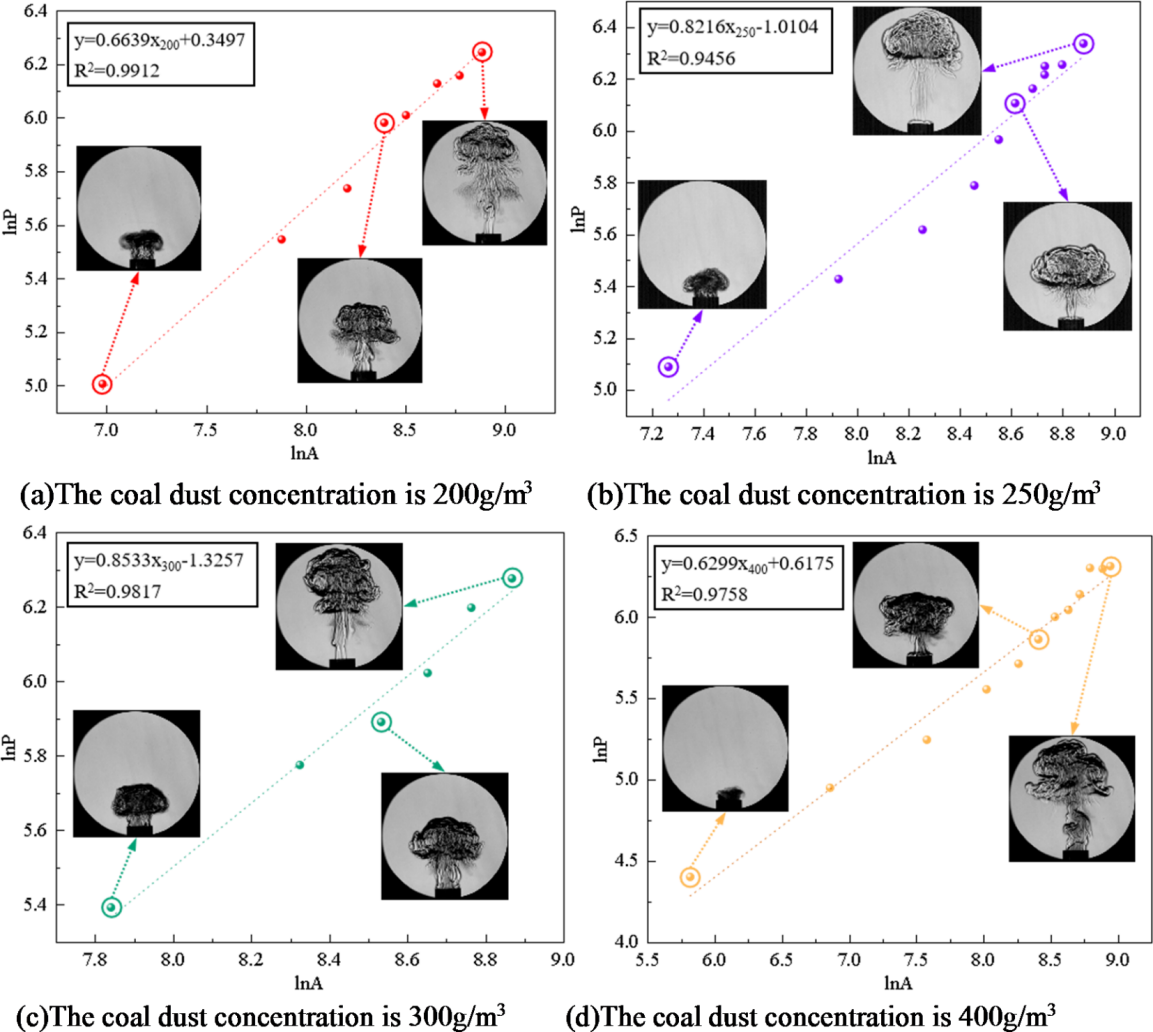

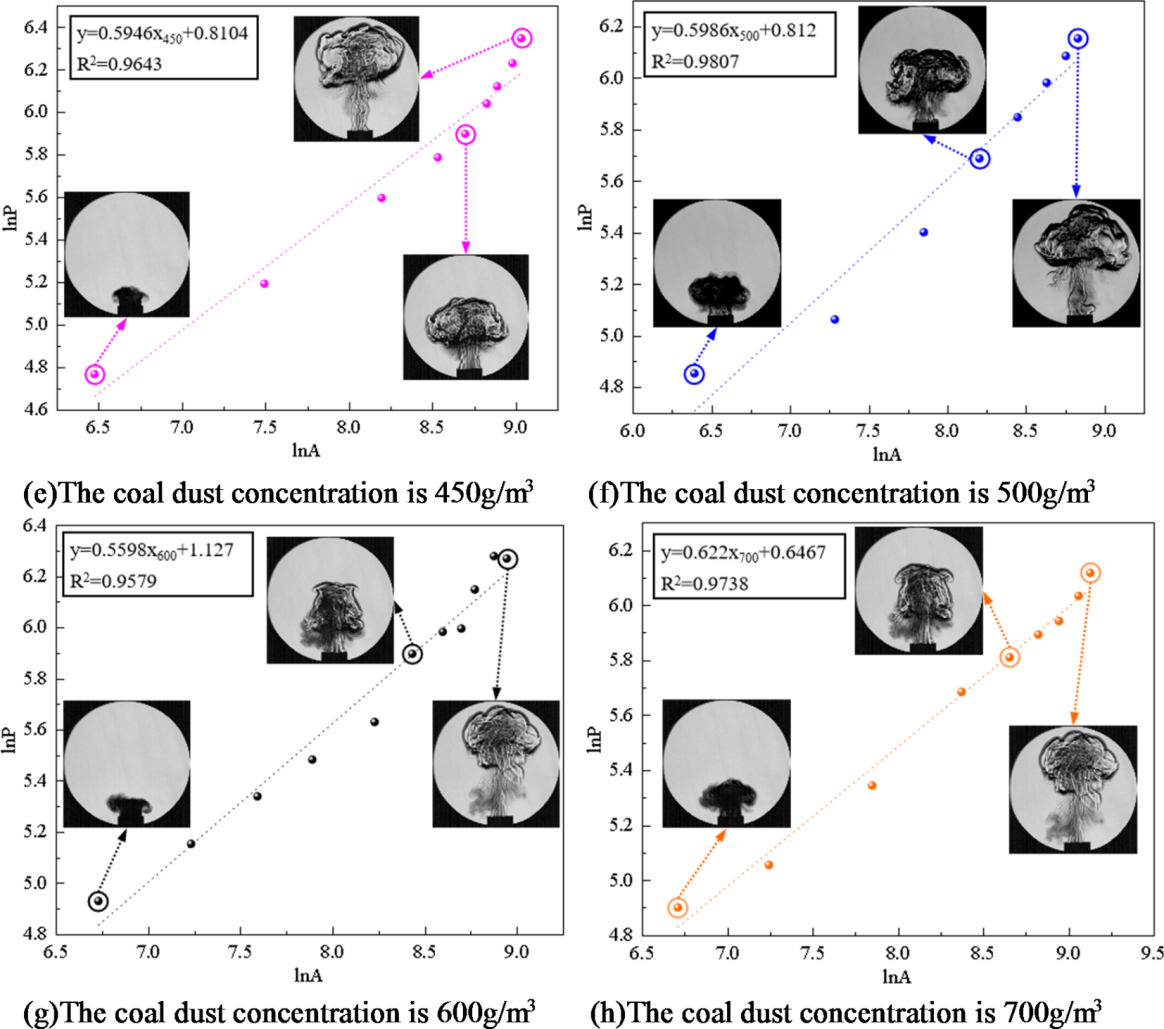
Flame fitting equation of coal dust particle size less than 53 μm at different concentrations.

To gain a deeper understanding of the flame’s geometric characteristics, the software imports the extracted parameters for “area *A*” and “perimeter *P*”. According to Eq. 3, the perimeter and area of the flame obtained in the experiment are logarithmically converted. Through logarithmic transformation, the nonlinear factors in the data are eliminated, which makes the subsequent analysis more accurate and reliable. Further processing yielded fitting equations for coal dust explosion flames at varying concentrations of two distinct coal dust particle sizes (particle size 53–75 μm, particle size less than 53 μm ), as seen in Figs. 8 and 9. Therefore, the island fractal dimension D*f* of coal dust explosion flame can be calculated, which indicates that the flame distribution has fractal characteristics.

**Fig. 10:**
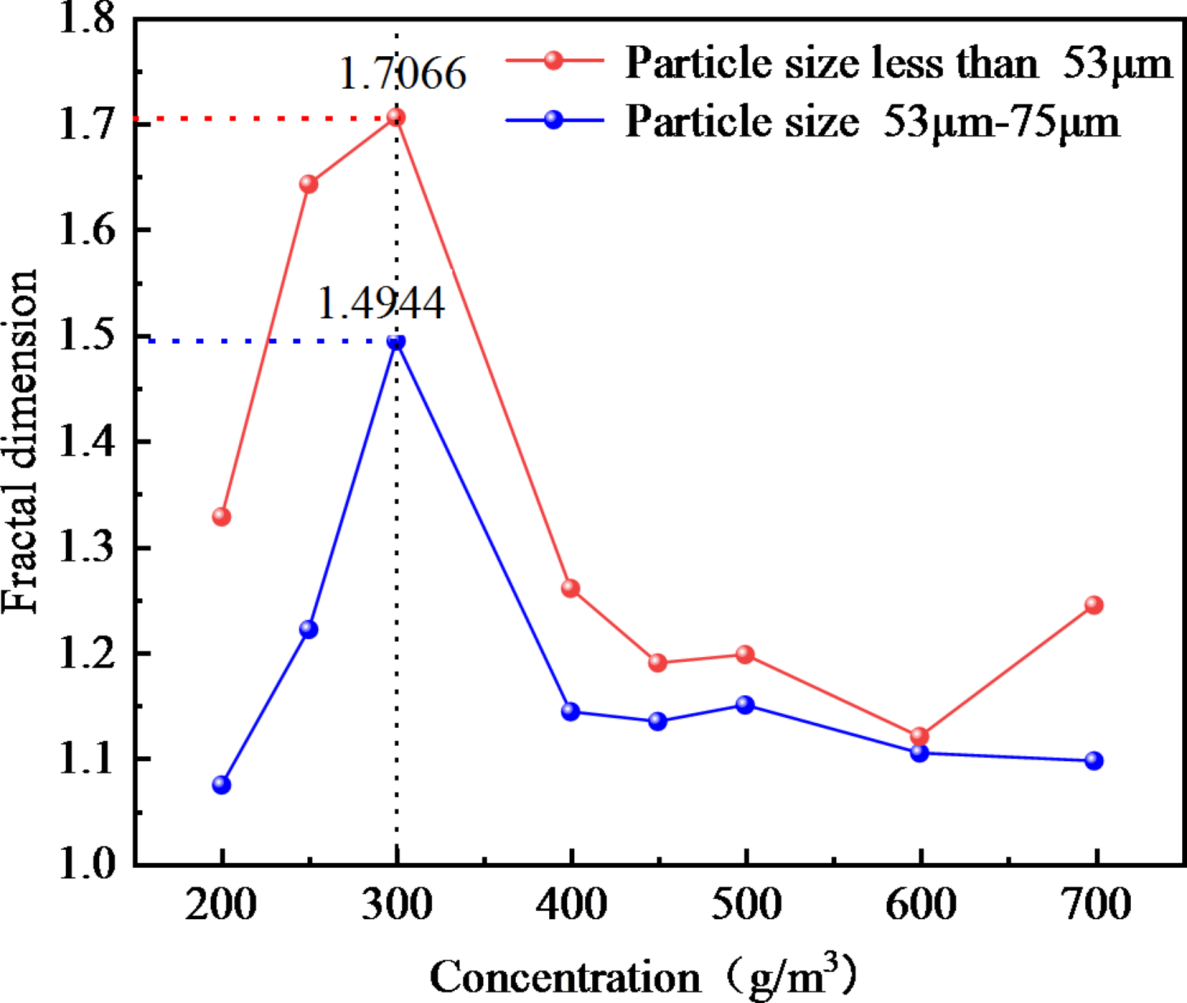
The fractal dimension of coal dust flame.

The Slit Island Method is used to measure the fractal dimension of flames by examining their scale relationship between surface area and perimeter. Fractal dimension serves as a measurement index for describing branching, shape, and complexity characteristics of flames. Through the experimental results, the fitting equation is obtained. According to the slope of the equation, the fractal dimension of coal dust explosion flame is further calculated, and the fractal dimension curve of coal dust flame is drawn as shown in Fig. 10.

Figure 10 illustrates that when the particle size of coal dust is certain, the fractal dimension initially increases and then decreases as the concentration of coal dust increases. When the initial concentration of coal dust is less than 300 g/m^3^, as the initial concentration increases, the combustion material and energy supply of the flame are more sufficient, resulting in the flame to present more branches and detailed structures, promoting the instability of the flame and increasing the surface area of the flame. This increased fractal structure and detail causes an increased fractal dimension of the flame. The increase of coal dust concentration will also lead to the acceleration of combustion rate, which will also affect the flame shape and further increase its fractal dimension. Therefore, when describing the flame form according to the island method, as the concentration increases, the fractal dimension of the flame increases, indicating that the flame shape is more complex and has more branches, and the flame combustion is unstable.

The fractal dimension peaks at an initial coal dust concentration of 300 g/m^3^. During this phase, the flame exhibits its highest complexity and branching compared to other concentrations.

For initial coal dust concentrations exceeding 300 g/m^3^, the fractal dimension decreases as the concentration increases. This occurs due to a restriction in combustion within Hartmann tube caused by higher coal dust concentration, leading to inadequate fuel and oxygen supply alongside challenging fuel-oxygen mixing. Insufficient mixing will lead to incomplete combustion reaction. In this case, the flame may become more limited and incomplete, lacking branches and detailed structures, resulting in a decrease in fractal dimension. The increase of coal dust concentration results in the change of flame heat transfer characteristics. The increase of concentration may lead to a decrease in flame temperature, which in turn reduces thermal radiation and flame surface area, thereby reducing the fractal dimension. With the increase of coal dust concentration, the filling effect of coal dust particles in space becomes more significant. The high concentration of coal dust cloud will fill the space, and the interaction between coal dust particles will be strengthened, resulting in more dense aggregation between particles. This aggregation will lead to a decrease in the gap between the particles in the coal dust cloud, making the spatial structure of the whole system more compact and more evenly distributed, resulting in a decrease in the fractal dimension. The increase of coal dust concentration leads to the change of flame heat transfer characteristics, which leads to the decrease of flame temperature, and reduces thermal radiation and flame surface area, thus reducing the fractal dimension. With the increase of coal dust concentration, the filling effect of coal dust particles in space becomes more significant. High concentration of coal dust clouds will fill the space, and the interaction between coal dust particles will be strengthened, resulting in more dense aggregation between particles. This aggregation will lead to the decrease in the gaps between particles in the coal dust cloud, making the spatial structure of the whole system more compact and more evenly distributed, resulting in a decrease in the fractal dimension.

Figure 10 illustrates that larger coal dust particle sizes result in a reduction in the fractal dimension of the flame. Smaller coal dust particles with reduced particle sizes possess a higher surface area-to-volume ratio, leading to a more intricate and efficient combustion process. The increased specific surface area of smaller particles enhances their susceptibility to effective diffusion between oxygen and fuel, accelerating the attainment of combustion temperature, initiating combustion promptly, and ensuring a more thorough and efficient combustion process. Furthermore, coal dust particles of smaller sizes exhibit increased susceptibility to effective diffusion, leading to accelerated burning rates that contribute to a diverse and intricate flame formation process. The accelerated and dynamic nature of this combustion process results in a flame shape that is irregular and intricate, consequently elevating the fractal dimension of the flame. The combustion properties exhibited by smaller-sized coal dust particles contribute to enhanced geometric complexity within the flame structure, ultimately resulting in an augmented fractal dimension of the flame.

Larger coal dust particles require additional time and energy to initiate combustion, influencing the formation process of flames in their vicinity. Coal dust particles with larger sizes may exhibit slower combustion rates, leading to a milder and uniform flame shape that diminishes the fractal dimension of the flame. The fractal dimension is commonly employed to characterize the geometric attributes of intricate structures like flames. Nonetheless, the combustion behavior exhibited by larger-sized coal dust particles results in flame geometrical features aligning more closely with regular structures, thereby causing a reduction in the fractal dimension of the flame. Consequently, as coal dust particle size increases, larger particles necessitate additional energy and time for combustion, resulting in a more uniform flame shape and a decrease in fractal dimension.

In conclusion, the front surface and internal structure of a coal dust explosion flame exhibit distinct fractal properties. The linear correlation of the ln*P* - ln*A* function is strong, indicating an interaction between fractal dimension and flame propagation. The wrinkles on the flame’s leading edge alter the interface area between coal dust and the flame, consequently affecting the chemical reaction rate. Through a fractal geometry lens, a higher fractal dimension results in a more intricate and convoluted surface profile at the flame front, allowing for increased participation of coal dust particles in combustion reactions and subsequently enhancing heat release rates. Simultaneously, there is an escalation in flame turbulence intensity along with an increase in observable cellular structures on its surface.

### Analysis of cell structure of coal dust cloud

**Fig. 11:**
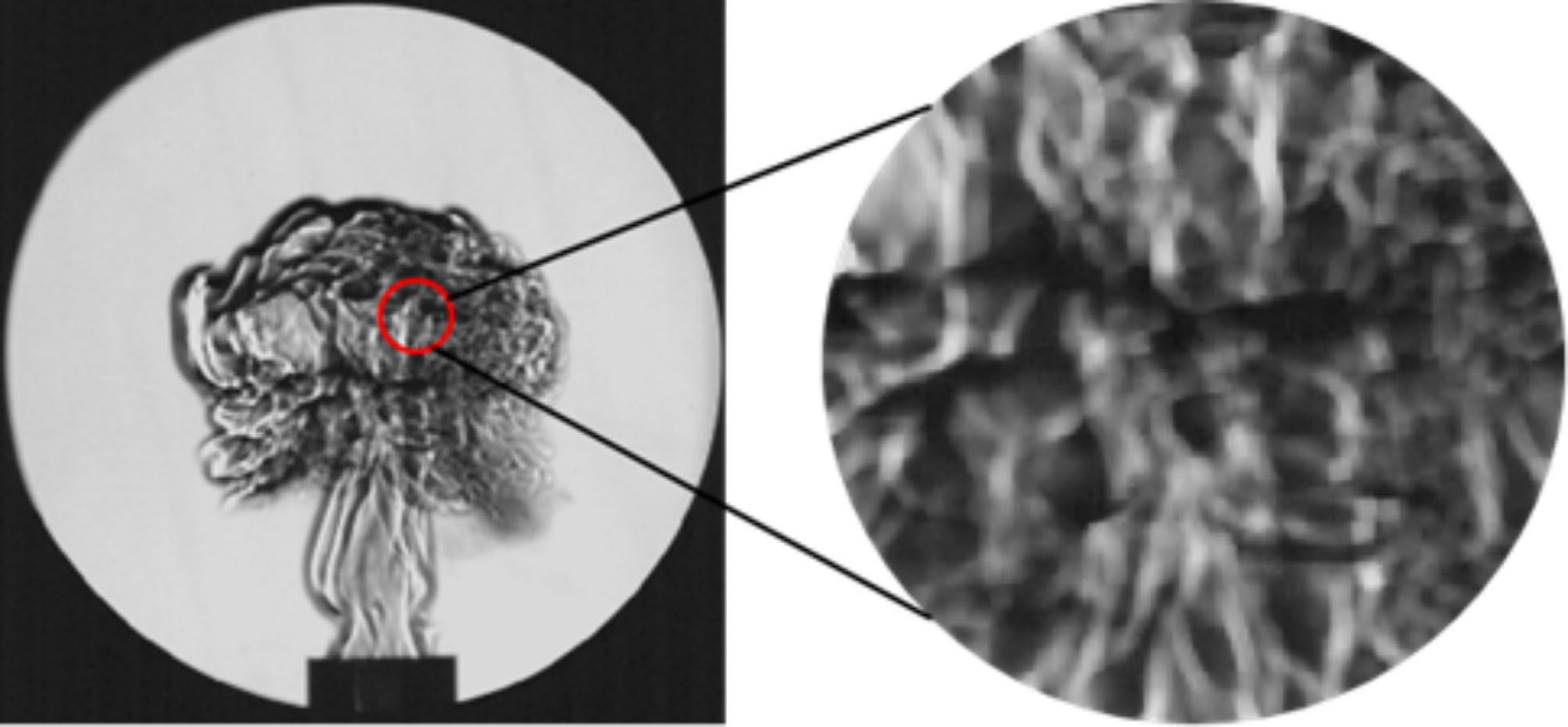
Cell-shaped flame of coal dust explosion under schlieren image.

During the development of the flames, flame is usually stretched caused by the tangential velocity gradient of the flame surface^34^. The surface is wrinkled and gradually develops into multiple cells of different sizes. As can be seen from Fig. 11, the coal dust explosion flame exhibits a unique cellular structure. In the process of coal dust combustion, the reaction does not ideally occur only at a single flame front, but is widely distributed on multiple surfaces where coal dust particles can undergo gaseous decomposition, so that the flame surface is completely covered by the cell structure. This cell-shaped flame not only increases the flame front area and accelerates the flame propagation speed, but also may lead to greater peak pressure or a wider range of combustion or detonation.

**Fig. 12:**
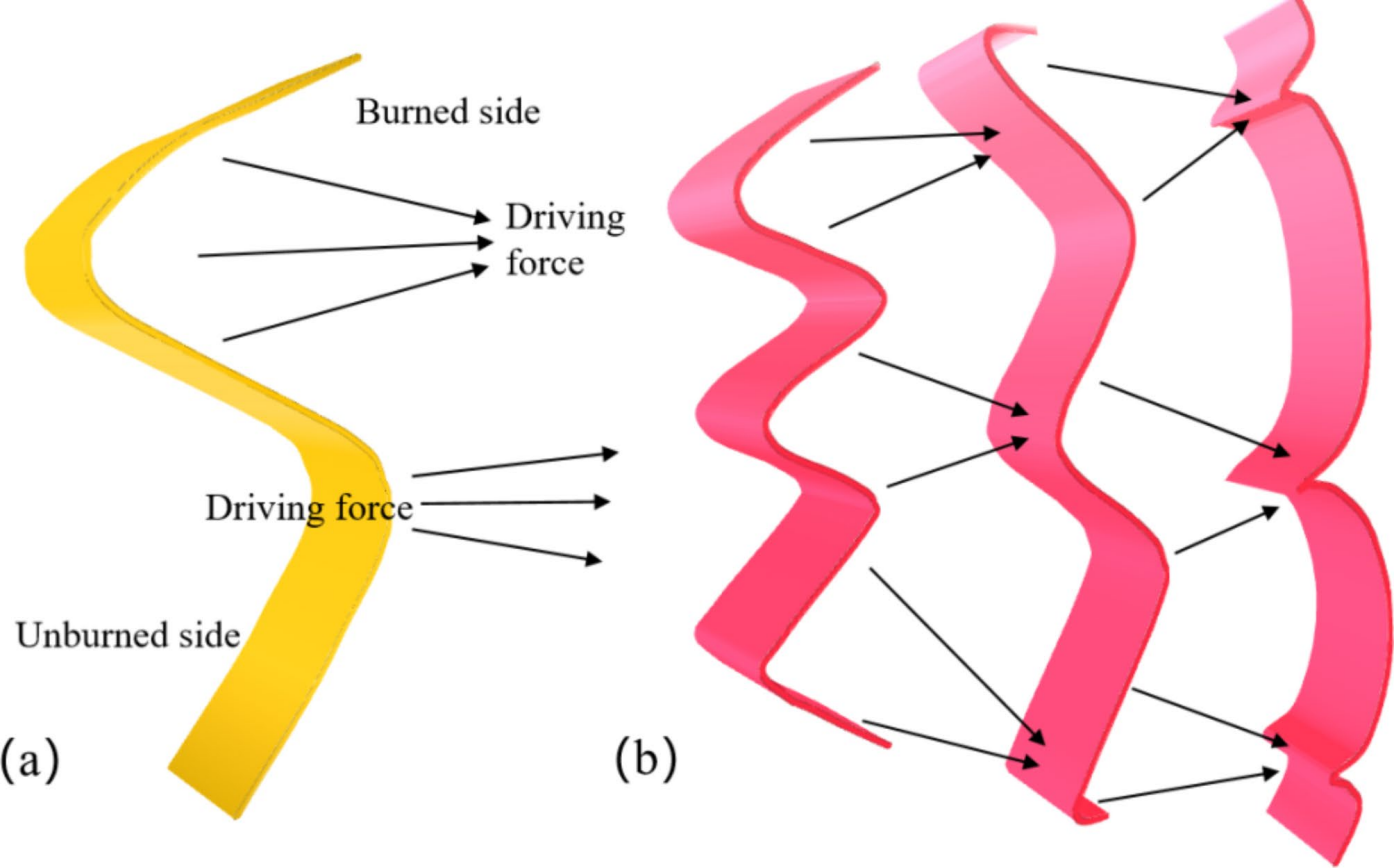
Fluid dynamic instability mechanism.

The instability of flame cell structure of coal dust explosion is mainly affected by many factors. Among them, hydrodynamic instability is one of the key factors. Hydrodynamic instability refers to a phenomenon caused by the difference in density between the unburned region and the combustion interface, also known as hydraulic instability or Darrieus-Landau (DL) instability^35–37^. In the combustion process, the hydrodynamic instability caused by the thermal expansion effect is particularly prominent, especially in the exothermic reaction stage. Figure 12 illustrates the mechanism of hydrodynamic instability in detail. When the thermal expansion gas passes through the flame, it causes the acceleration of local flow and the change of thrust. The convex part of the flame front will produce greater thrust due to stronger thermal expansion. The concave part is weakened by the thermal expansion effect, and the thrust is reduced accordingly. This change in the spatial distribution of thrust further promotes the growth of disturbance on the surface of the flame front. Figure 12(b) illustrates the dynamic evolution of instability during flame wave propagation. Due to the widening of the bottom of the concave protrusion area, the position of the peak point remains almost unchanged, the concave area becomes flat, and the opposite side disturbance disappears. This process makes the unburned and burned areas unstable due to fluid stretching and flame stretching, resulting in unstable flame propagation^38^, which divides the surface of the flame front into a series of independent cellular structures.

**Fig. 13:**
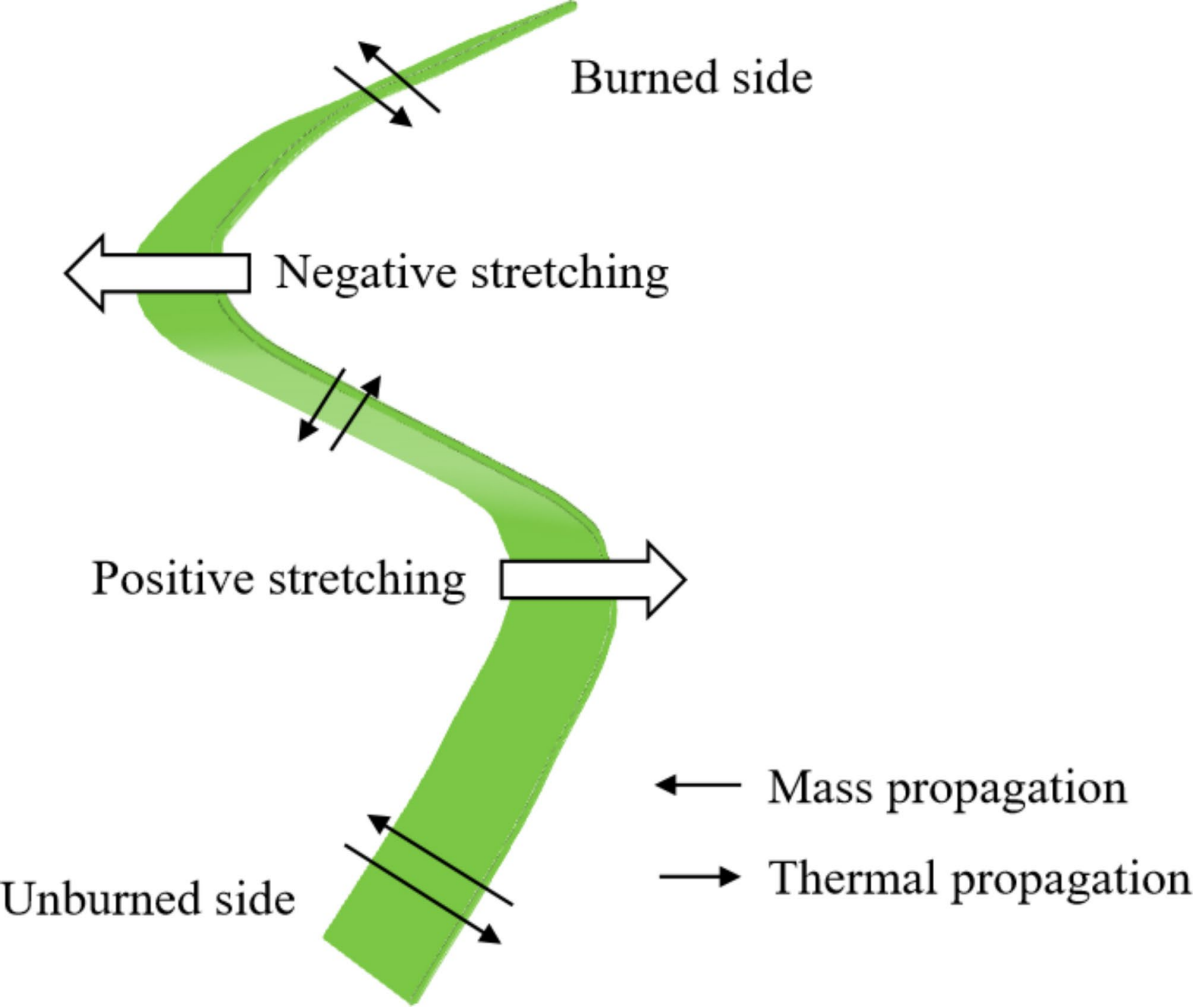
Unequal diffusion instability.

Unequal diffusion instability also plays a role in the stability of the cellular flame structure. In addition to heat diffusion, the flame cell expands with mass diffusion. The Lewis number (Le) is the ratio of the thermal diffusivity of the mixture to the mass diffusivity of the poor reactant, where the poor reactant refers to the relative lack of material in the reactant. In the fuel-rich region, the mass diffusion rate is the mass diffusion rate of oxygen. While in the fuel-poor region, the fuel mass diffusion rate is taken. The Lewis number can be used to characterize the thermal-mass diffusion instability of the flame. The higher its value, the stronger the perturbation of flame surface and the stronger the flame instability. When Le > 1, the heat loss caused by thermal diffusion exceeds the energy input of mass diffusion. As shown in Fig. 13, the positive stretching surface is wrapped with the reactants, and the heat dissipation of the flame to the convex part of the unburned zone is greater. The temperature of the combustion gas is greater than the adiabatic flame temperature, and the flame propagation speed decreases. In contrast, the reverse stretching surface is opposite. The front is wrapped with unreacted substances, the heat dissipation of the flame to the unburned zone is small, the temperature of the combustion gas is less than the adiabatic flame temperature, and the combustion speed increases. At this time, the positive and negative stretching of the flame surface cancels each other out, the flame convex part tends to be stable, and the flame wrinkle tends to be stable. When Le < 1, the heat loss of thermal diffusion is less than the energy input of mass diffusion. The heat dissipation of the front towards the convex direction of the unburned zone is smaller and the propagation speed is accelerated. At the same time, the reverse stretching surface propagates faster to the burned zone. The positive and negative stretching of the flame surface is more and more serious, and the flame wrinkle tends to be unstable. In the forward stretching process of the outward expansion of the cellular flame, due to the different factors such as the concentration, pressure and temperature of the frontal reactants in the flame diffusion process, the heat and mass diffusion of Le < 1 occurs in some areas, and the flame instability produces a concave and convex structure. The flame surface is covered with irregular cells, forming an unequal diffusion instability.

Buoyancy instability also affects the cell structure of the flame. Under the action of buoyancy, the density of the burned area is smaller than that of the unburned area, and the flame moves to the high-density unburned area. However, buoyancy instability is only reflected when the combustion speed is slow. It often occurs when the combustion time is long enough, the combustion volume is large enough or the combustion limit, and its influence is negligible in most studies.

In summary, the instability of the flame cell structure of coal dust explosion is mainly caused by the combined action of hydrodynamic instability and unequal diffusion instability. These unstable developments will change the flame front structure, from the wrinkles on the surface of the flame sphere in the early stage to the wrinkles. The division gradually develops into cell bodies of different sizes, and then the cell body gradually becomes more and more. Finally, the flame volume continues to increase, and the flame structure changes drastically.

## Conclusions

Based on the self-built coal dust explosion test device, this paper uses a high-speed camera and a schlieren to record the dynamic process of flame propagation at the vertical outlet position of Hartmann. The temporal and spatial evolution characteristics of coal dust explosion flames with different concentrations and particle sizes were studied by fractal dimension.

1. The flame shape of Hartmann outlet is mushroom cloud, and the front surface of coal dust flame and the internal structure of flame have obvious fractal characteristics.
2. When the particle size of coal dust is constant, the fractal dimension increases first and then decreases with the increase of concentration.
3. The increase of coal dust particle size leads to the decrease of flame fractal dimension. When the particle size of coal dust is less than 53 μm and the initial concentration is 300 g/m^3^, the flame fractal dimension reaches the maximum value of 1.71.
4. The coal dust explosion flame has a unique cell structure, and the instability of its structure is mainly affected by the interaction of hydrodynamic instability and unequal diffusion instability.

## Data Availability

The datasets used and analysed during the current study available from the corresponding author on reasonable request.
